# Bioinformatics analysis reveals the characteristics of immune microenvironment in major depressive disorder and vitiligo

**DOI:** 10.1371/journal.pone.0352672

**Published:** 2026-07-08

**Authors:** Yayun Xiang, Hongmei Deng, Yanyue Wang, Ning Yan, Yang Lei

**Affiliations:** 1 Department of Neurology, University-Town Hospital of Chongqing Medical University, Chongqing, China; 2 Medical Sciences Research Center, University-Town Hospital of Chongqing Medical University, Chongqing, China; Wuhu Hospital Affiliated to East China Normal University, CHINA

## Abstract

**Background:**

Major depressive disorder (MDD) and vitiligo often occur together, worsening patient outcomes. However, the shared pathogenic mechanisms remain unclear.

**Methods:**

This study applied integrated bioinformatics to identify shared candidate markers for MDD and vitiligo. Public transcriptomic datasets from the GEO database were analyzed for differential expression. Protein-protein interaction (PPI) networks were constructed using the STRING database. Shared differentially expressed genes (DEGs) underwent GO and KEGG functional enrichment analyses. Three machine-learning algorithms were applied to select candidate biomarker genes. Additionally, immune infiltration analysis was quantified through ssGSEA and a TF-miRNA network was constructed via NetworkAnalyst platform. Single-gene GSEA further explored pathways linked to the biomarker in both diseases.

**Results:**

Differential expression analysis and PPI network construction suggest the involvement of 14 hub genes potentially linked to both MDD and vitiligo. Functional enrichment analyses indicate their putative roles in immune processes and inflammatory responses. Machine learning further prioritized three key genes: *EXOSC7*, *KLRG1*, and *MAPK14*. Immune infiltration analysis revealed distinct patterns of inferred immune enrichment signatures, and the TF-miRNA network highlighted the complexity of the regulatory landscape. Preliminary validation suggests *MAPK14* as a potential candidate gene warranting further investigation in MDD and vitiligo.

**Conclusion:**

This study provides preliminary evidence suggesting that immune dysregulation and inflammatory activation may be interconnected in MDD and vitiligo. *MAPK14* represents a potential candidate marker for their comorbidity. These findings primarily serve to generate hypotheses regarding shared mechanisms and prioritize targets for subsequent experimental validation.

## 1. Introduction

Major depressive disorder (MDD) significantly impairs individuals’ psychological well-being and social functioning [1]. Epidemiological evidence indicates that depression affects over 332 million people globally and is predicted to become a leading contributor to the global burden of disease by 2030 [2]. Concurrently, vitiligo presents another challenge. This chronic autoimmune disease is characterized by progressive melanocyte loss and skin depigmentation [3]. It heavily impacts patients’ social activities and mental health [4]. The psychosocial stress factors such as embarrassment from their damaged appearance are also associated with the onset or exacerbation of vitiligo [5]. Strikingly, up to 62% of vitiligo patients suffer from comorbid MDD [6], potentially leading to poorer clinical responses to therapy.

Previous research has shown that the melanocortin system, particularly the key receptors MC1R and MC4R, is dysregulated in patients with vitiligo [7]. Additionally, comparative transcriptome analysis indicated that melanocytes exhibit a gene expression profile reminiscent of their neural crest origin, including synapse-associated proteins and neuropeptide receptors [8]. Melanocytes and neurons share not only an embryonic origin but also key signaling molecules (e.g., endothelins, FGFs) and pathways, positioning melanocytes as a pertinent model for studying nervous system disorders [9]. Moreover, melanocytes display neuroendocrine characteristics and are sensitive to emotion-related neural signals, such as stress‑induced neuropeptides [10]. Together, these findings point to a common physiopathological mechanism underlying the comorbidity of vitiligo and MDD, suggesting that their association is rooted in shared biology rather than psychosocial factors alone.

Dysfunction of melanocytes is a core link in vitiligo pathogenesis. Kingo et al. have revealed that the expression of multiple genes within key intracellular melanogenesis pathways, notably the cAMP/PKA, Wnt, and MAPK pathways (e.g., *MITF*, *LEF1*, *p38*, *PIK3CB*, *RPS6KB1*), are significantly dysregulated in vitiligo patients [11]. Furthermore, autoimmune dysregulation, oxidative stress, and the aberrant inflammatory signaling are also involved in the pathogenesis of vitiligo [3,12]. Similarly, immune and inflammatory hypotheses have been proposed to account for the pathophysiology of MDD [13]. Individuals with vitiligo frequently experience chronic stress, anxiety, and diminished self-esteem [6]. Such stress can activate microglia and astrocytes, promoting a pro-inflammatory condition linked to depressed behaviors [14]. Clinical studies have reported that the levels of inflammatory cytokines were elevated in the plasma of MDD patients [15,16]. These factors also mediate the immunity and inflammatory response in the skin [17]. Peripheral inflammatory cytokines can cross the blood-brain barrier and modulate neurons and glial cells, influencing brain function and well-being [18,19]. Thus, immune dysregulation and systemic inflammation may increase the risk of depression in vitiligo. Although the exact mechanisms are still unclear, shared features of immune dysregulation and inflammatory activation appear to underpin both diseases. Elucidating the shared mechanisms underlying both diseases is a crucial step. Identifying novel candidate biomarkers for their coexistence could enable earlier detection and more targeted interventions.

In this study, we integrated genes related to MDD and vitiligo from GEO databases and applied multiple bioinformatics approaches, especially machine learning and immune infiltration analysis, to reveal the shared pathogenic mechanisms and potential biomarkers for their comorbidity.

## 2. Methods

### 2.1. Data acquisition and processing

In this study, gene expression profiles for MDD and vitiligo were sourced from the publicly available Gene Expression Omnibus (GEO) database (www.ncbi.nlm.nih.gov/geo). For MDD, the GSE98793 dataset was utilized, which includes 192 whole blood samples: 64 from healthy controls, 128 from patients with MDD, using the GPL570 platform for sequencing. For vitiligo, the GSE65127 and GSE53146 datasets were integrated using the R package ‘sva’ to correct batch effects. The combined datasets included 15 healthy samples and 15 vitiligo samples, using the GPL570 and GPL14951 platforms for sequencing. External validation was performed using the independent MDD dataset GSE52790 and vitiligo dataset GSE80009. All data were standardized, and probe annotations were normalized. We note that the small number of vitiligo participants restricts the scope of this analysis and its general findings.

### 2.2. Identification of differentially expressed genes

Differentially expressed genes (DEGs) between disease and control groups were identified using the R package ‘limma’, applying a threshold of |log2FC| > 0 and P-value < 0.05. Results were visualized via ‘ggplot2’ and ‘pheatmap’. Overlapping DEGs between MDD and vitiligo were extracted and visualized using a Venn diagram.

### 2.3. Construction of protein-protein interaction (PPI) network

The co-expressed DEGs were imported into the STRING website (v12.0, [href:https://string-db.org/]https://string-db.org/), and then a PPI network was constructed with a confidence score threshold of 0.4. The network was visualized in Cytoscape (v3.9.1), and hub genes were identified using the plugin CytoHubba-MCC algorithm.

### 2.4. Functional enrichment analysis of hub genes

To elucidate the biological functions and mechanisms of the hub genes, Gene Ontology (GO) and Kyoto Encyclopedia of Genes and Genomes (KEGG) pathway enrichment analyses were conducted with the ‘clusterProfiler’ R package (P-value < 0.05). Additionally, we evaluated whether specific signaling pathways were enriched in different samples. Gene Set Enrichment Analysis (GSEA) was performed using the c2.cp.all.v2022.1.Hs.symbols.gmt gene set.

### 2.5. Acquisition of key genes by machine-learning algorithms

Given the limited sample size of vitiligo cases, we employed a consensus-based strategy to mitigate overfitting and enhance the robustness of feature selection. Specifically, we integrated the results from three distinct machine learning algorithms: LASSO logistic regression, Random Forest (RF), and Support Vector Machine Recursive Feature Elimination (SVM-RFE). Only features consistently selected by all three algorithms were retained for further analysis. We performed LASSO logistic regression using the ‘glmnet’ R package. The key parameters were set as family = ‘binomial’, nfolds = 5. A fivefold cross-validation method was employed to determine the optimal λ value to constrain the feature set, and the minimum λ value was selected as the final parameter. Additionally, SVM-RFE was applied using the ‘e1071’ R package to refine the selection of informative genes. Similarly, RF was employed using the ‘randomForest’ R package, constructing 1000 decision trees to assess variable importance. We ranked genes based on their importance scores for selecting potential markers. The top-ranked genes identified by RF, along with those selected by LASSO and SVM-RFE, formed the consensus list. We combined the results of three machine-learning algorithms and visualized the overlapping candidate markers using UpSet diagrams.

### 2.6. Immune infiltration analysis

We estimated the relative enrichment of immune cells using Single-Sample Gene Set Enrichment Analysis (ssGSEA) via the ‘GSVA’ R package, evaluating 28 distinct immune cell types based on specific gene sets [20]. It should be noted that these ssGSEA scores represent inferred immune signatures from bulk RNA-seq data, rather than direct measurements of actual immune cell abundances. In addition, Spearman correlation analysis was used to assess the relationships between key genes and immune cell enrichment scores.

### 2.7. Gene-gene and TF-miRNA co-regulatory networks

The gene-gene interaction network of the candidate biomarkers was constructed using the GeneMANIA online database([href:https://genemania.org/]https://genemania.org/) to identify potential functional associations and underlying mechanisms. Transcription factor (TF)-miRNA co-regulatory networks were constructed using the NetworkAnalyst platform and visualized in Cytoscape (v3.9.1).

### 2.8. Validation of key gene signatures

To evaluate the discriminatory ability of the key genes, we generated receiver operating characteristic (ROC) curves using the ‘pROC’ package to assess their performance and calculated the area under the curve (AUC). Two independent GEO datasets (GSE52790 and GSE80009) were utilized as external validation sets to examine the consistency and stability of these findings.

### 2.9. Identification of candidate compounds

The Drug Signature Database (DSigDB) on the Enrichr platform ([href:https://maayanlab.cloud/Enrichr/]https://maayanlab.cloud/Enrichr/) was used to predict candidate compounds for key genes closely associated with MDD and vitiligo [21].

### 2.10. Statistical analysis

Raw data processing and statistical analyses have been conducted using R version 4.3.3, and significant differences between groups were subsequently determined using Student’s t-test, with p < 0.05 considered statistically significant.

## 3. Results

### 3.1. Identification of DEGs associated with MDD and vitiligo

**Fig 1:**
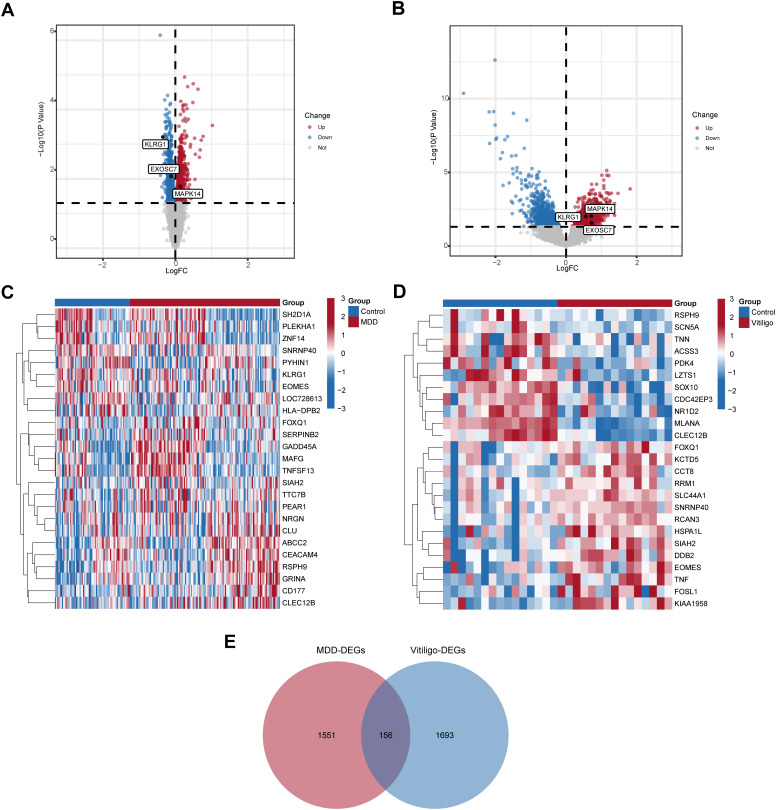
Identification of DEGs in MDD and vitiligo. **(A, B)** Volcano map of DEGs in MDD (A) and vitiligo (B). **(C, D)** Heatmaps of hierarchical clustering of the top 25 DEGs in MDD (C) and vitiligo (D). **(E)** Identification of 156 co-expressed genes by overlapping the DEGs in MDD and vitiligo.

After normalizing all datasets, a total of 1707 DEGs were screened in the MDD dataset GSE98793, including 818 upregulated and 889 downregulated genes (Fig 1A). In the combined vitiligo datasets, a total of 1849 DEGs were screened, with 960 upregulated and 889 downregulated genes (Fig 1B). Volcano plots were used to show the distribution of DEGs in each dataset. The lists of DEGs were available in S1 and S2 Tables. Heatmaps of the top 25 DEGs in MDD and vitiligo were presented in Fig 1C and Fig 1D, respectively. Further intersecting the DEGs from MDD and vitiligo revealed 156 co-expressed genes for analysis (Fig 1E).

### 3.2. Construction of PPI network and identification of hub genes

**Fig 2:**
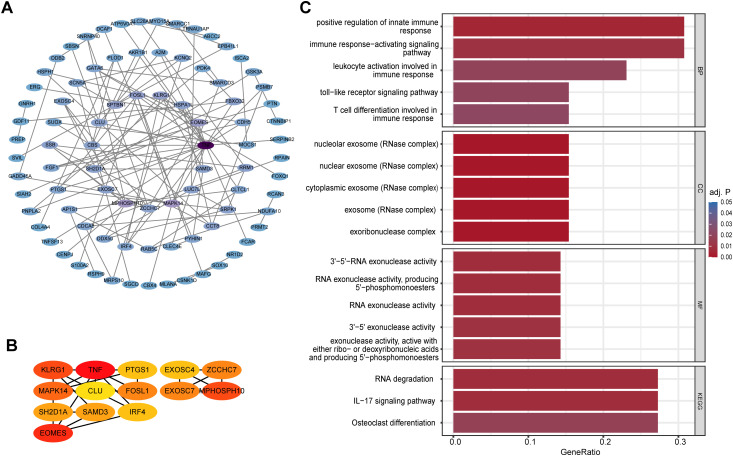
Screening hub genes and functional enrichment analysis of hub genes. **(A)** Protein-protein interaction network of common DEGs in MDD and vitiligo. **(B)** The 14 hub genes with the highest connectivity based on the CytoHubba-MCC algorithm. **(C)** GO and KEGG enrichment analysis of 14 hub genes.

A PPI network was constructed based on 156 co-expressed genes using the STRING database and Cytoscape software (v3.9.1). This network comprised 87 nodes and 113 edges, corresponding to genes and their interactions, respectively (Fig 2A). Based on the CytoHubba-MCC algorithm, the top 14 hub genes were selected: *KLRG1*, *TNF*, *PTGS1*, *EXOSC4*, *ZCCHC7*, *MAPK14*, *CLU*, *FOSL1*, *EXOSC7*, *MPHOSPH10*, *SH2D1A*, *SAMD3, IRF4*, and *EOMES* (Fig 2B).

### 3.3. Functional annotation and enrichment analysis of hub genes

GO and KEGG analyses were performed to evaluate the biological roles of the 14 hub genes. In the biological process (BP) category, significant enrichment was observed in positive regulation of innate immune response, immune response-activating signaling pathway, leukocyte activation involved in immune response, toll-like receptor signaling pathway, T cell differentiation involved in immune response, highlighting the role of immune and inflammatory processes in the development of both diseases. In the cellular component (CC) category, the 14 hub genes were primarily involved in nucleolar exosome (RNase complex), cytoplasmic exosome (RNase complex), nuclear exosome (RNase complex), exosome (RNase complex), and exoribonuclease complex, indicating the possible contribution of RNA metabolism disorders to disease pathogenesis. In the molecular function (MF) category, the significantly enriched terms were 3’-5’-RNA exonuclease activity, 3’-5’ exonuclease activity, RNA exonuclease activity, RNA exonuclease activity, producing 5’-phosphomonoesters, and exonuclease activity, active with either ribo-or deoxyribonucleic acids and producing 5’-phosphomonoesters. These molecular functions underscore the importance of regulating the biological processes involved in both diseases. KEGG pathway analysis further complemented the findings by identifying functional enrichments such as RNA degradation, IL-17 signaling pathway, and osteoclast differentiation. Finally, the results of the GO and KEGG enrichment analysis was visualized through bar chart (Fig 2C).

### 3.4. Identification of key genes by integrating machine learning algorithms

**Fig 3:**
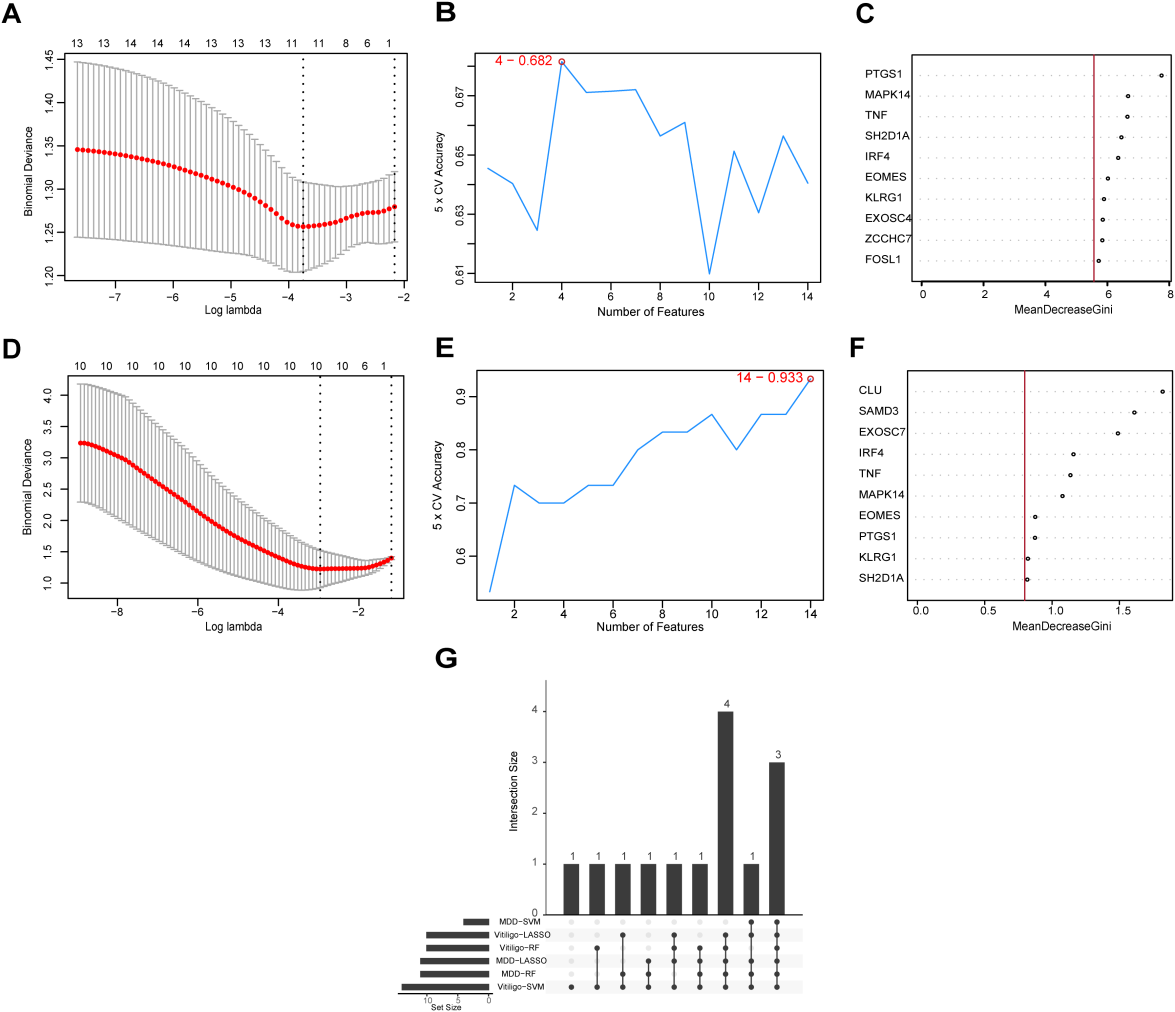
Identification of key genes by different machine learning algorithms. **(A, D)** Feature selection using the LASSO regression method in MDD (A) and vitiligo (D). **(B, E)** Feature selection using the SVM‑RFE in MDD (B) and vitiligo (E). **(C, F)** Gene importance ranking derived from random forest analysis in MDD (C) and vitiligo (F). **(G)** UpSet diagram illustrating the intersection of genes identified by the three machine‑learning algorithms.

To further identify common biomarkers in MDD and vitiligo, we applied three machine learning algorithms (LASSO, SVM-RFE, and RF) as feature selection toolsbased on 14 hub genes. In the MDD dataset, the LASSO algorithm narrowed down the list to 11 candidate genes (Fig 3A). The SVM‑RFE method identified a subset of genes based on recursive feature elimination (Fig 3B). Subsequently, the RF algorithm ranked the genes according to their importance scores, highlighting 11 top‑ranked candidates (Fig 3C). In the vitiligo dataset, the LASSO algorithm screened 10 characteristic genes (Fig 3D), while SVM‑RFE selected 14 genes (Fig 3E). The RF algorithm also provided a ranked list of genes for vitiligo (Fig 3F). The complete lists of genes selected by these machine learning algorithms are provided in S3 and S4 Tables. Ultimately, we intersected the results of the machine learning methods and identified three common key genes: *EXOSC7*, *KLRG1*, and *MAPK14* (Fig 3G), which may serve as preliminary candidate markers for further mechanistic studies in MDD and vitiligo.

### 3.5. Immune infiltration analysis of the key genes

**Fig 4:**
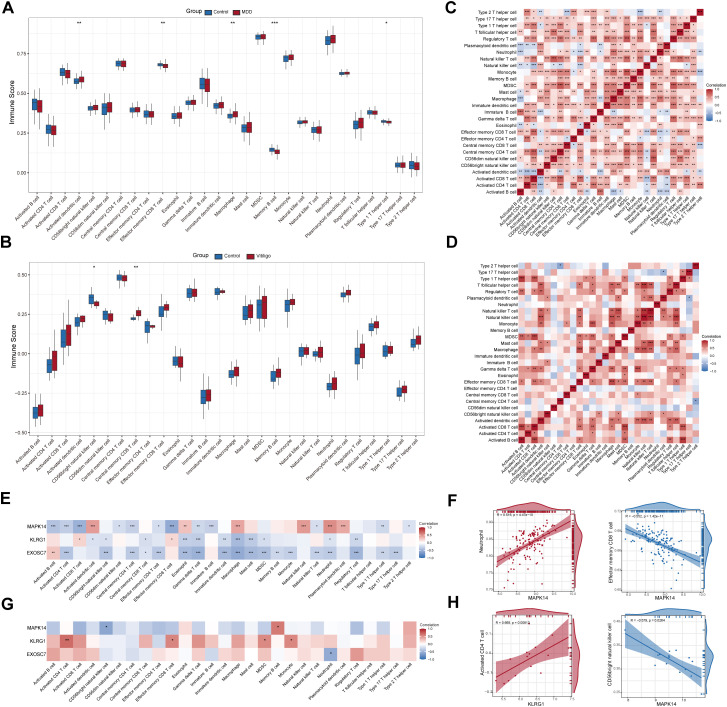
ssGSEA analysis of immune infiltration. **(A, B)** Boxplot of immune cell enrichment scores inferred by ssGSEA in MDD (A) and vitiligo (B). **(C, D)** The correlation heatmaps between immune cell enrichment scores in MDD (C) and vitiligo (D). **(E, F)** The correlation between key genes (*EXOSC7*, *KLRG1*, and *MAPK14*) and immune cell enrichment scores in MDD. **(G, H)** The correlation between key genes (*EXOSC7*, *KLRG1*, and *MAPK14*) and immune cell enrichment scores in vitiligo. * P-value < 0.05, ** P-value < 0.01, *** P-value < 0.001.

The ssGSEA algorithm was used to compare the immune cell enrichment scores of 28 immune cell types between cases and healthy controls, and boxplots illustrated the differences of these scores in patients with MDD and vitiligo (Figs 4A-B). In the MDD dataset, enrichment scores for activated dendritic cells and macrophages were significantly elevated (P-value < 0.05), while those for effector memory CD8 + T cells, memory B cells, and type 1 T helper cells were significantly reduced (P-value < 0.05). In the vitiligo dataset, enrichment scores for central memory CD8 + T cells were significantly elevated, and those for CD56bright natural killer cells were significantly reduced (P-value < 0.05).

In addition, we performed Spearman rank analysis to explore the relationships between candidate marker genes and immune cell enrichment scores. The correlation heatmaps showed strong intercellular correlations in MDD and vitiligo, respectively (Figs 4C-D). Analysis of the three key genes revealed distinct correlation patterns with these immune scores. *KLRG1* was positively correlated with effector memory CD8 + T cells, while *EXOSC7* was negatively correlated with neutrophils in both diseases (Figs 4E and 4G). *MAPK14* showed the strongest positive correlation with neutrophils (cor = 0.516, P-value < 0.05) and the strongest negative correlation with effector memory CD8 + T cells in the MDD dataset (cor = −0.552, P-value < 0.05) (Fig 4F). In the vitiligo dataset, *KLRG1* was correlated with activated CD4 + T cells (cor = 0.668, P-value < 0.05), and *MAPK14* was most negatively correlated with CD56bright natural killer cells scores (cor = −0.579, P-value < 0.05) (Fig 4H).

### 3.6. Co-regulatory networks of genes and TF–miRNA interactions

**Fig 5:**
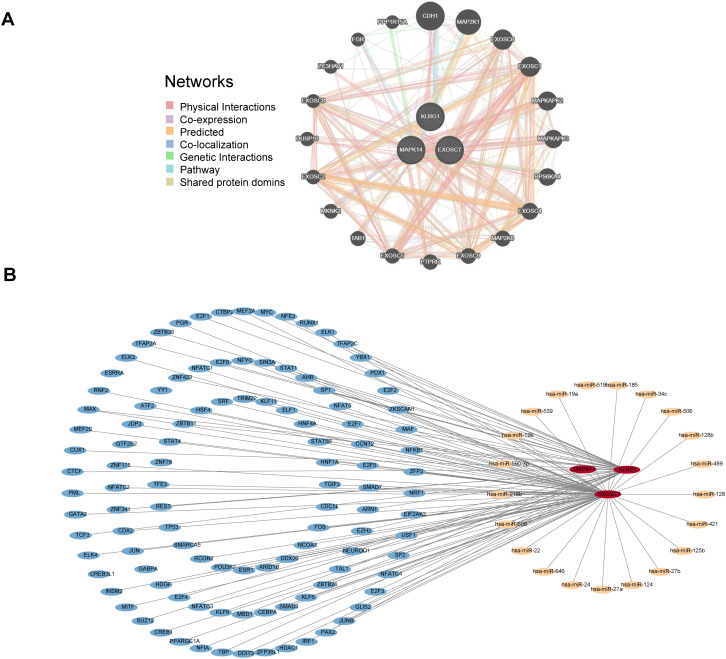
The interaction network of key genes. **(A)** The interaction network of key genes via GeneMANIA. **(B)** Co-regulatory network of TF-miRNA and three key genes.

The GeneMANIA database was used to construct the interaction network of the three key genes. Fig 5A showed the network involving the biomarkers and their most connected neighboring genes, including physical interactions, predicted associations, co‑expression, co‑localization, genetic interactions, shared pathways, and protein domain similarity. Furthermore, a TF–miRNA co‑regulatory network containing 21 miRNAs and 111 TFs was also built, illustrating how these molecules may jointly regulate the expression of the three key genes (Fig 5B).

### 3.7. Validation of key gene expression and performance

**Fig 6:**
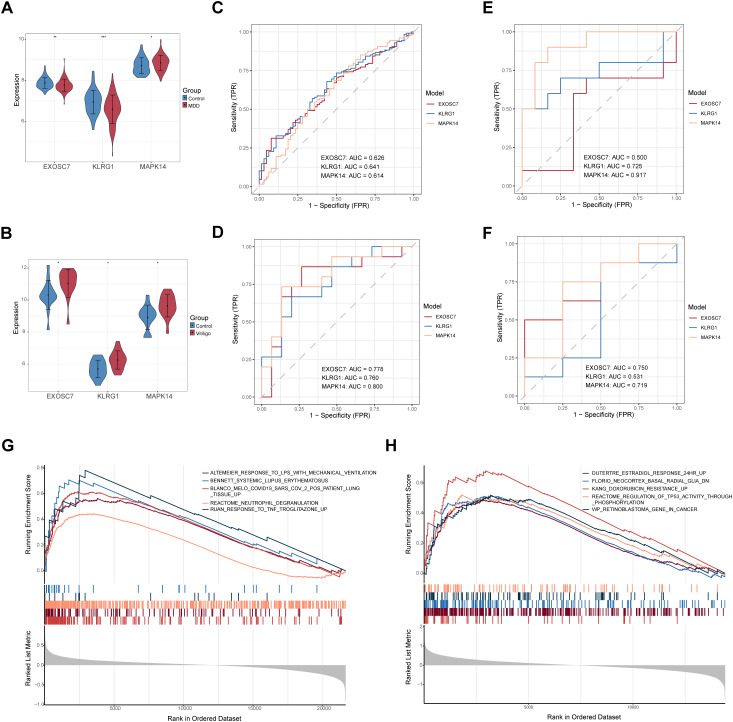
Validation of key genes and single‑gene GSEA analysis. **(A, B)** Validation of the expression of key genes in the MDD dataset (A) and vitiligo dataset (B) based on T-test. * P-value < 0.05, ** P-value < 0.01, *** P-value < 0.001. **(C, D)** ROC curves of key genes to assess their discriminatory ability in MDD (C) and vitiligo (D). **(E, F)** ROC curves of key genes to evaluate their performance in the external validation datasets GSE52790 (E) and GSE80009 (F). **(G, H)** Representative enriched pathways identified by single‑gene GSEA for *MAPK14* in MDD (G) and vitiligo (H).

The expression patterns of the three key genes were evaluated across the training dataset and two external validating datasets. Expression levels were first confirmed in the training dataset. The results showed that *MAPK14* was upregulated in both diseases (Figs 6A-B). Subsequently, ROC analysis was conducted to assess the discriminatory ability of these genes between disease and control groups. In the MDD dataset, the AUC values ranged from 0.614 to 0.641 (Fig 6C). In the vitiligo dataset, they ranged from 0.760 to 0.800 (Fig 6D). In the validation sets, the performance remained consistent, with AUC values ranging from 0.725 to 0.917 for dataset GSE52790 (Fig 6E) and from 0.531 to 0.750 for dataset GSE80009 (Fig 6F). Notably, *MAPK14* demonstrated the most stable performance, maintaining an AUC greater than 0.6 across all datasets, suggesting it as a robust candidate for further mechanistic investigation.

### 3.8. Single-gene GSEA analysis

We performed single-gene GSEA of the candidate biomarker *MAPK14* to identify related pathways in MDD and vitiligo datasets. In the MDD dataset, BLANCO_MELO_COVID19_SARS_COV_2_POS_PATIENT_LUNG_TISSUE_UP, ALTEMEIER_RESPONSE_TO_LPS_WITH_MECHANICAL_VENTILATION, REACTOME_NEUTROPHIL_DEGRANULATION, RUAN_RESPONSE_TO_TNF_TROGLITAZONE_UP, BENNETT_SYSTEMIC_LUPUS_ERYTHEMATOSUS, and other immune inflammation-related pathways were significantly enriched (Fig 6G). In the vitiligo dataset, significant pathways included KANG_DOXORUBICIN_RESISTANCE_UP, DUTERTRE_ESTRADIOL_RESPONSE_24HR_UP, FLORIO_NEOCORTEX_BASAL_RADIAL_GLIA_DN, WP_RETINOBLASTOMA_GENE_IN_CANCER, and REACTOME_REGULATION_OF_TP53_ACTIVITY_THROUGH_PHOSPHORYLATION (Fig 6H). Detailed results of the top 50 most significant pathways of single-gene GSEA were provided in S6 and S6 Tables. Crucially, both enriched pathways showed functions related to immune inflammation, suggesting it may be a shared pathophysiological mechanism in MDD and vitiligo.

### 3.9. Identification of drugs associated with key genes

DSigDB was used to search for drug-gene interactions. For *MAPK14*, 124 interacting compounds were retrieved. The top five candidate compounds are listed in Table 1. It is important to note that these predictions are based on computational algorithms. While they provide a starting point for future research, the biological effects of these compounds remain hypothetical and require rigorous experimental validation.

**Table 1:** Prediction of key genes-Related Drugs.

Term	P-value	Adjusted P-value	Odds Ratio	Combined Score	Genes
Tyrphostin AG 1478 TTD 00011607	0.00165	0.03151	999.35000	6403.33481	MAPK14
Go 6976 TTD 00008282	0.00180	0.03151	908.45455	5741.92129	MAPK14
Talmapimod CTD 00004435	0.00195	0.03151	832.70833	5196.55351	MAPK14
Doramapimod TTD 00007708	0.00225	0.03151	713.67857	4351.68576	MAPK14
B-Octylglucoside TTD 00002290	0.00240	0.03151	666.06667	4018.41623	MAPK14
TBTO CTD 00000264	0.01076	0.03662	140.32394	635.91604	EXOSC7
15-delta prostaglandin J2 HL60 DOWN	0.01448	0.04079	103.65104	438.95980	EXOSC7
latamoxef HL60 DOWN	0.01768	0.04413	23.37690	94.32808	EXOSC7, MAPK14
colchicine HL60 DOWN	0.01937	0.04685	77.00775	303.70362	EXOSC7
tetryzoline HL60 DOWN	0.02011	0.04748	74.11567	289.51923	EXOSC7

## 4. Discussion

MDD has become one of the most prevalent comorbidity in patients with vitiligo because the disfiguring appearance significantly affects their physical and mental health [22]. Complex crosstalk between neurotransmitters and hormones links the central nervous and cutaneous systems, and mental health disorders may trigger or exacerbate skin conditions via the brain-skin axis [23]. Genetic factors have been explored in the pathogenesis of MDD and vitiligo. The heritability of MDD involves multiple neurotransmitter system genes, with SNPs in *CCKAR*, *DRD1*, *DRD2*, and *HTR2C* linked to MDD risk [24]. The *WFS1* gene has also been proven to increase risk of emotional disorders [25]. Notably, both *WFS1* and *CCKAR* reside within the 4p15-p16 locus, a genomic region implicated in mental disorders, vitiligo susceptibility, and other immune-mediated diseases [26]. Interestingly, MDD exhibits a marked seasonal pattern [27], which animal models attribute to elevated brain CCK and 5-HT_2_ receptor densities under summer-like conditions [28]. Studies links solar UV exposure to vitiligo onset [29], pointing to shared environmental triggers across both disorders. Thus, depression and vitiligo extend beyond mere comorbidity, involving bidirectional pathological crosstalk between the two diseases.

Previous studies have explored candidate biomarkers for MDD or vitiligo separately. Transcriptome analysis of vitiligo has shown that the dysregulated gene sets are implicated in key processes including cell death, survival, signaling, inflammation, and oxidative stress [30]. However, shared mechanisms and biomarkers in their comorbidity remain unclear. To our knowledge, we report the first combined bioinformatics analysis of these two disorders. By integrating public transcriptomic datasets, 156 co-expressed genes were identified. Subsequent GO and KEGG enrichment analyses revealed biological processes and pathways implicated in their shared pathology. GO enrichment highlighted immune response and inflammatory pathways, along with cellular components and molecular functions linked to RNA metabolic processes. Given that vitiligo is recognized as an inflammatory disorder, studies have shown that elevated IL‑22 levels are associated with progressive vitiligo [31]. Similar elevated levels of inflammatory response were also observed in MDD [15].

KEGG analysis of the shared gene set revealed marked enrichment in several pathways, most notably the IL‑17 signaling pathway. It raises the possibility that this pro-inflammatory pathway may represent a common molecular feature linking the two conditions. Prior studies support a role for IL-17 in vitiligo pathogenesis, where it has been associated with reduced melanocyte viability and dysfunctional melanin production [32,33]. Similarly, clinical studies link increased levels of IL-17 with MDD [34]. IL-17A modulates despair-like behaviors by regulating GABAA receptor α2 subunit-mediated inhibitory synaptic transmission in the hippocampus [35]. It also can promote neuroinflammation by activating glial cells and increasing blood-brain barrier permeability [36–38]. Based on these observations and our bioinformatics results, we hypothesize that IL-17 signaling may be a candidate mechanism underlying the comorbidity of the two diseases. However, our data only establish an associative relationship rather than a causal one. The precise mechanism remains speculative and requires further investigation. Future studies including measurements of serum IL-17 in comorbid patients and preclinical mechanistic models are needed to validate this hypothesis.

Elevated inflammatory mediators in MDD and vitiligo point to immune activation as a driver of onset and progression [15,17]. In the MDD cohort, we observed increased enrichment scores for activated dendritic cells and macrophages. While dendritic cells are known to promote antigen presentation and T cell priming, and their activation is linked to the production of IL‑6 and TNF‑α implicated in depressive symptoms [39–41], our data do not directly measure these functional outputs. Similarly, M1 macrophages, recognized as key mediators of inflammation, have been associated with depression mechanisms [19,42]. Conversely, reduced enrichment scores for effector memory CD8 + T cells, memory B cells, and Th1 cells were noted, suggesting potential adaptive immune impairment potentially linked to compromised rapid recall responses and defense [43]. In vitiligo, higher enrichment scores for central memory CD8 + T cells were observed, consistent with a shift toward stronger adaptive immunity. These cells, which reside in lesional and peri‑lesional skin, are capable of recognizing melanocyte antigens and mediating melanocyte apoptosis via IFN‑γ and TNF [44]. Enrichment scores for CD56bright NK cells were decreased, possibly disrupting innate immune surveillance needed to maintain melanocyte integrity [45]. Spearman correlation analysis revealed that the three key genes were strongly correlated with various immune cell enrichment scores. The findings suggest that these key genes may be involved in shaping the immune microenvironment and thereby influence MDD in vitiligo patients. Further studies are needed to clarify causal pathways and the interplay between immune cell dysfunction and these key genes.

In this study, we identified *MAPK14* as a candidate gene that may be involved in both MDD and vitiligo. This result came from the combination of multiple machine learning methods and was checked in two independent GEO datasets. However, the sample size of the discovery cohort was small, and all findings are based on bioinformatics analysis rather than experimental or clinical data. The mechanistic role of *MAPK14* in clinical outcomes for patients with MDD and vitiligo remains largely unexplored. *MAPK14* is a member of the mitogen-activated protein kinase family. The p38-MAPK pathway is known to respond to endotoxins, pro-inflammatory cytokines (e.g., TNF‑α, IL‑1), and heat stress [46]. Previous studies have shown that *MAPK14* can regulate inflammatory molecules like IL‑1β, IL‑6, and TNF‑α, and has been linked to neuroinflammation as well as melanocyte apoptosis [47–50]. In our analysis, *MAPK14* was consistently upregulated, suggesting that shared inflammatory pathways may contribute to both conditions. Furthermore, single-gene GSEA for *MAPK14* suggested enhanced immunological activity in MDD, particularly involving neutrophil degranulation. Clinical evidence indicates that neutrophil counts and inflammatory responses are often elevated during active depressive episodes [51]. Interestingly, genes linked to COVID‑19 were also observed in our analysis. Studies have reported that viral infection disrupts neutrophil degranulation and alters myeloperoxidase, and ultimately reducing peripheral B‑cell and T‑cell numbers [52]. These findings imply that abnormal degranulation is linked to immune cell depletion. Neutrophil degranulation shapes disease progression not only through direct inflammation but also by modulating immune cell activity. Overall, given the limited sample size, our results should be interpreted as hypothesis‑generating. While we identify *MAPK14* as a potential key gene, these findings alone cannot establish a causal relationship. Future studies involving larger, well‑characterized clinical cohorts and basic laboratory experiments are essential to validate the regulatory mechanisms of *MAPK14*.

Several limitations should be noted. First, data were obtained from the GEO repository, which may carry biases from original studies, such as sample size variation and population heterogeneity. These factors can limit the generalizability of our findings. Second, the sample size for vitiligo was relatively small, which represents a key limitation of this study. Although we employed consensus machine‑learning strategies and external validation to mitigate overfitting, the small discovery cohort may still limit the generalizability of the findings and could potentially lead to false positives. Therefore, the identified genes should be interpreted as exploratory candidates pending validation in larger, independent clinical cohorts. Third, to capture subtle transcriptional changes and identify candidate genes for downstream validation, we employed a permissive log threshold and nominal p-values. However, this approach may carry a risk of false positives. Finally, further experimental studies including in vitro and in vivo assays are warranted to validate the biomarkers and elucidate the underlying mechanisms.

## 5. Conclusions

In conclusion, our study identified *MAPK14* as a preliminary candidate biomarker for MDD and vitiligo, suggesting potential shared molecular pathways between these distinct conditions. By integrating differential expression profiling, PPI networks, and machine learning, we proposed the role of *MAPK14* in both diseases. Functional enrichment analyses indicated potential involvement of key genes in relevant signaling cascades, which requires further verification. Furthermore, immune infiltration analysis and exploration of TF–miRNA regulatory networks offered a tentative view of the underlying biological mechanisms. Together, these findings provide a computational basis for future research into the pathophysiology of MDD and vitiligo. We propose *MAPK14* as a preliminary candidate marker, but stress that it requires extensive experimental and clinical validation before any clinical use. These results are best viewed as generating new hypotheses and guiding future studies, rather than providing a finished diagnostic tool.

## Supporting information

S1 Table Full list of DEGs between MDD and control. (XLSX)

S2 Table Full list of DEGs between vitiligo and control. (XLSX)

S3 Table Candidate biomarkers identified by machine learning algorithms in MDD. (XLSX)

S4 Table Candidate biomarkers identified by machine learning algorithms in vitiligo. (XLSX)

S5 Table Top 50 significant enrichment results of single-gene GSEA in MDD. (XLSX)

S6 Table Top 50 significant enrichment results of single-gene GSEA in vitiligo. (XLSX)
