# Supplementary material for: Bioinformatics analysis reveals the characteristics of immune microenvironment in major depressive disorder and vitiligo
Source: PLoS One. 2026 Jul 8;21(7):e0352672. doi: 10.1371/journal.pone.0352672 (PMC13345404; doi:10.1371/journal.pone.0352672)
Supplement: Attachment — Submitted filename: Response to Reviewers.docx [file pone.0352672.s008.docx]

**Response Letter to Reviewers' Comments**

Dear Editor Dr. Liu and Reviewers,

We are very grateful for the valuable comments and constructive suggestions from you and the reviewers on our manuscript entitled "*Bioinformatics analysis reveals the characteristics of immune microenvironment in major depressive disorder and vitiligo*" (PONE-D-26-06215). Based on the feedback received, we have carefully revised the manuscript accordingly. All modifications have been **highlighted in red** in the revised version for easy identification. In response to the comments, we provide a point-by-point reply below. Once again, we sincerely appreciate you and the reviewers' input, which has greatly helped to improve the quality of our work. Here are our responses to the reviewers’ comments one-by-one.

**Response to reviewer #1:**

**Comment 1: The rationale for linking vitiligo and MDD is not convincingly supported. Consider discussing peptides, neuropeptides, the melanocortin system, or melanocytes (PMID: 17651944 IF: 4.0 Q1, PMID: 25545474 IF: 2.6 Q2). Melanocytes possess a gene expression profile very similar to that of neurons.**

**Response:** We thank the reviewer for this important comment. We have incorporated the suggested references (PMID: 17651944 and PMID: 25545474) into the revised manuscript. In the Introduction section, we added a discussion on the similarity between melanocytes and neurons, emphasizing their shared gene expression profiles and the role of the melanocortin system and neuropeptides in linking vitiligo and MDD. This suggested a possible shared biological mechanism underlying both vitiligo and MDD. These modifications have been incorporated into the manuscript (pages 3-4, lines 70-77) as follows:

**References added in the revised manuscript:**

7. Kingo K, Aunin E, Karelson M, Philips M-A, Rätsep R, Silm H, et al. Gene expression analysis of melanocortin system in vitiligo. J Dermatol Sci. 2007;48: 113–122. doi:10.1016/j.jdermsci.2007.06.004

8. Reemann P, Reimann E, Ilmjärv S, Porosaar O, Silm H, Jaks V, et al. Melanocytes in the skin--comparative whole transcriptome analysis of main skin cell types. PloS One. 2014;9: e115717. doi:10.1371/journal.pone.0115717

**Comment 2: The pathogenesis of vitiligo can be better explained, along with more relevant cellular pathways such as the one in (PMID: 18514490 IF: 4.0 Q1).**

**Response:** We appreciate the reviewer’s comment. We have expanded the description of vitiligo pathogenesis in the Introduction section. Specifically, we incorporated the cellular pathway from PMID: 18514490, highlighting the role of melanocyte destruction. This provides a more comprehensive background for our study. The text has been revised accordingly on pages 4, lines 82-85:

**References added in the revised manuscript:**

11. Kingo K, Aunin E, Karelson M, Rätsep R, Silm H, Vasar E, et al. Expressional changes in the intracellular melanogenesis pathways and their possible role in the pathogenesis of vitiligo. J Dermatol Sci. 2008;52: 39–46. doi:10.1016/j.jdermsci.2008.03.013

**Comment 3: Meanwhile, mood disorders have a substantial genetic component, involving many neuropeptides (PMID: 15927089 IF: 3.7 Q1, PMID: 15473915 IF: 3.7 Q1).**

**Response:** We have added a discussion on the genetic basis of mood disorders, citing PMID: 15927089 and PMID: 15473915. We now describe the involvement of neuropeptides and genetic variants in MDD, which supports the rationale for exploring shared molecular mechanisms with vitiligo. We have amended the manuscript as requested on pages 17, lines 345-349:

**References added in the revised manuscript：**

24. Kõks S, Nikopensius T, Koido K, Maron E, Altmäe S, Heinaste E, et al. Analysis of SNP profiles in patients with major depressive disorder. Int J Neuropsychopharmacol. 2006;9: 167–174. doi:10.1017/S1461145705005468

25. Koido K, Kõks S, Nikopensius T, Maron E, Altmäe S, Heinaste E, et al. Polymorphisms in wolframin (WFS1) gene are possibly related to increased risk for mood disorders. Int J Neuropsychopharmacol. 2005;8: 235–244. doi:10.1017/S1461145704004791

**Comment 4: Additionally, the seasonality of MDD and vitiligo, as well as the seasonal nature of anxiety and mood disorders (PMID: 10721682IF: 3.3 Q2), should be discussed. This seasonality has been demonstrated in laboratory animals and is related to peptide and peptidergic transmission. More discussion and exploration of this topic are needed.**

**Response:** We are grateful for the reviewer’s valuable observation. We have included a new part in the Discussion section addressing the seasonality of both MDD and vitiligo. We cited PMID: 10721682 and discussed how seasonal changes may influence the occurrence of diseases, adding depth to the mechanistic link. The changes have been implemented in the manuscript (page 17, lines 351-356):

**References added in the revised manuscript：**

28. Kõks S, Männistö PT, Bourin M, Shlik J, Vasar V, Vasar E. Cholecystokinin-induced anxiety in rats: relevance of pre-experimental stress and seasonal variations. J Psychiatry Neurosci JPN. 2000;25: 33–42.

**Comment 5: The authors overlooked a study with genome-wide transcriptomics on vitiligo patients (PMID: 27047918). This should be addressed at minimum. Also, other gene expression studies on clinically relevant vitiligo samples, such as (PMID: 18717682), warrant discussion.**

**Response:** We thank the reviewer for this important comment. We have cited both PMID: 27047918 and PMID: 18717682 in the revised manuscript. We compared our findings with these studies and highlighted how our work extends prior transcriptomic analyses to the context of MDD comorbidity. These modifications have been incorporated into the manuscript (pages 17, lines 357-360 and page 18, lines 367-369) as follows:

**References added in the revised manuscript：**

30. Reimann E, Kingo K, Karelson M, Reemann P, Vasar E, Silm H, et al. Whole Transcriptome Analysis (RNA Sequencing) of Peripheral Blood Mononuclear Cells of Vitiligo Patients. Dermatopathology. 2014;1: 11–23. doi:10.1159/000357402

**References added in the revised manuscript：**

31. Rätsep R, Kingo K, Karelson M, Reimann E, Raud K, Silm H, et al. Gene expression study of IL10 family genes in vitiligo skin biopsies, peripheral blood mononuclear cells and sera. Br J Dermatol. 2008;159: 1275–1281. doi:10.1111/j.1365-2133.2008.08785.x

**Response to reviewer #2:**

**Comment 1:** **The differential expression threshold is too permissive and likely inflates false-positive findings. The DEG strategy should be revised using a more appropriate threshold, ideally with adjusted P values and a nontrivial fold-change cutoff.**

**Response:** We sincerely thank the reviewer for raising this important point. We acknowledge that the relatively permissive threshold may carry a risk of false positives. Our initial choice was motivated by the exploratory nature of this study. This relatively permissive threshold has been described in previous studies (e.g. PMID: 40369032, 38791423, 39419187) and was chosen to ensure that genes with smaller expression changes were not overlooked. While adjusted p-values are commonly used, the application of nominal p-values combined with downstream filtering strategies is also prevalent in exploratory analyses to identify more potential candidates for further validation. To control for false positives and ensure the reliability of our findings, we implemented the following multi-step validation strategy:

1. Rather than relying solely on DEGs, we integrated PPI networks and intersected results from three machine learning algorithms (LASSO, SVM-RFE, Random Forest). This step effectively filtered out potential unstable candidates.

2. External Validation: We tested the final key genes in two independent GEO datasets. They were confirmed in these external data. This step ensures that our findings are robust and not merely artifacts of the initial screening.

Regarding the reviewer’s concern, we have addressed this issue in the revised manuscript. We have added a statement in the "Limitations" section of the Discussion explicitly acknowledging the potential bias introduced by the permissive threshold and advising caution in the interpretation of the results.

These modifications have been incorporated into the manuscript (pages 21, lines 441-444) as follows:

**Comment 2: The sample size (especially for vitiligo) is quite small for machine-learning-based conclusions.**

**Response:** We thank the reviewer for this important comment regarding the sample size. We fully agree that the vitiligo group is relatively small, which limits the development of a standalone clinical prediction model. Given the sample size constraints, this study was designed as an exploratory investigation rather than a definitive model-building exercise. We used machine learning as a feature selection tool to prioritize candidate genes from differential expressed genes. To reduce the impact of small sample size, we applied three independent algorithms (LASSO, SVM‑RFE, Random Forest). Only genes selected by all three methods were retained. And internal stability was assessed using repeated cross‑validation. Most importantly, the selected genes were further evaluated in two independent GEO datasets (GSE52790, GSE80009). The consistent performance across datasets (e.g., *MAPK14*: AUC = 0.800 and 0.719) suggests a degree of reproducibility, although we acknowledge these results remain exploratory. This strategy is consistent with previous studies using small‑sample transcriptomic data to identify potential biomarkers (e.g., PMID: 39814853).

In the revised manuscript, we have stated that this study serves as a hypothesis-generating analysis. We have clarified the use of cross-validation in the Methods, emphasized the exploratory nature in the Results, and expanded the discussion on the limitations of small sample sizes in the Discussion section. Thank you for helping us improve the rigor of our work. The specific changes are as follows:

page 7 (lines 139 to 144, Methods section 2.5)

page 8 (lines 171 to 175, Methods section 2.8)

page 20 (lines 428 to 432, Discussion section)

page 21 (lines 436 to 441, limitations section)

**Comment 3: The claim that MAPK14 is a “diagnostic biomarker” is too strong, given only modest AUC values. These results are more consistent with a preliminary candidate marker than with a robust diagnostic biomarker. The conclusions and abstract should be toned down accordingly.**

Response: We sincerely thank the reviewer for highlighting this important issue. We completely agree that the term “diagnostic biomarker” overstates our findings, especially given the modest AUC values. Our study is an exploratory study, which serves to identify hypotheses rather than provide clinical diagnostic tools. To address this, we have carefully revised the manuscript as follows:

1.We have systematically replaced “diagnostic biomarker” with more cautious terms such as “potential candidate gene” or “preliminary marker” throughout the Abstract, Results, and Conclusion sections.

2.We have removed any strong claims about clinical application. We now state clearly that these findings are intended for “hypothesis generation” and require further experimental validation in clinical samples in the Abstract and Conclusion sections.

These changes ensure the conclusions accurately reflect the nature of a bioinformatics analysis study. The modifications particularly conclusion section have been clearly stated on page 21-22 (lines 449 to 461):

**Comment 4: The immune landscape is inferred using ssGSEA from bulk transcriptomic data, not directly measured. Therefore, statements implying actual immune cell abundance or mechanistic immunoregulation should be toned down.**

**Response:** We thank the reviewer for this important point. We agree that ssGSEA only estimates immune activity from gene data and does not directly measure real cell numbers. To address this, we have carefully revised the manuscript as follows:

1. We replaced “immune cell levels” with “immune cell enrichment scores” throughout the manuscript.

2. We toned down causality by removing strong causal verbs like “regulate,” “cause,” or “drive.” We now use careful phrases such as “associate with” or “correlate with.”

3. We added clear statements to clarify method limitation in the Methods sections specifying that these findings represent inferred immune signatures, not direct measurements of cell abundance.

These revisions make sure our conclusions match the limits of the bioinformatics method used. We have updated the Methods, Results, and Discussion to reflect these points. The changes have been implemented in the manuscript (page 7-8, lines 157-163, page 12, lines 254 to 262, page 18-19, lines 386 to 391):

Methods sections:

Results sections:

Discussion sections:

**Comment 5:**

**1) The IL-17 discussion is plausible and biologically interesting, but it extends substantially beyond what is directly demonstrated by the manuscript.**

**Response:** We sincerely appreciate the reviewer’s insightful comment regarding the IL-17 signaling pathway. We agree that the current study is based on bioinformatics analysis and does not directly demonstrate the molecular mechanisms of IL-17 signaling. Accordingly, in the revised manuscript, we have removed the discussion regarding the potential therapeutic implications of IL-17. We have also toned down the mechanistic claims and reframed the narrative to emphasize that IL-17 is a plausible hypothesis generated by our data, rather than a confirmed mechanism. These changes are mainly in the Discussion section (Lines 370-385; Page 18). We hope this revision addresses your concern.

**2) Similarly, the therapeutic framing around MAPK14 and candidate drugs identified through Enrichr/DSigDB should be presented more cautiously as hypothesis-generating.**

**Response:** We thank the reviewer for this important comment. Following the reviewer’s suggestion, we have carefully revised the discussion related to *MAPK14*. We now clearly state that our findings are exploratory and hypothesis‑generating, rather than evidence of therapeutic efficacy. We have rewritten the discussion section about *MAPK14* to present the results with greater caution. And we also have removed any overstated implications regarding drug targets and have emphasized that, due to the limited sample size and the computational nature of the study, further experimental and clinical validation is required. The revised text explicitly highlights the need for future functional studies to confirm the role of *MAPK14* in the comorbidity of MDD and vitiligo. The following revisions have been made to the manuscript (page 15, lines 332-337, page 19-21, lines 407-432):

Discussion sections:

Thank you again for your valuable time and insightful comments, which have significantly strengthened our manuscript.

We look forward to hearing from you soon.

Sincerely,

Yayun Xiang, Ph.D.

Department of Neurology, University-Town Hospital of Chongqing Medical University

Email: 800238@hospital.cqmu.edu.cn

Date: May 14, 2026
